# Pathological buying on the rise? Compensative and compulsive buying in Poland in the pre- and (Post-)pandemic times

**DOI:** 10.1371/journal.pone.0298856

**Published:** 2024-03-21

**Authors:** Grzegorz Adamczyk

**Affiliations:** Institute of Sociology, Catholic University of Lublin, Lublin, Poland; University of Ghana, GHANA

## Abstract

The study concerns the development of compensative and compulsive buying in Poland comparing the results of three waves of a cross-sectional study conducted before and at the end of the COVID-19 pandemic. Six predictors of susceptibility to compensative and compulsive buying are in focus: materialism, self-esteem, gender, age, frequency of online shopping, and experience of the COVID-19 pandemic. However, the importance of the first four predictors in explaining compensative and compulsive buying is already very well described in the literature, while the novelty consists in the predictive model including the variables that describe frequency of online shopping and negative experiences related to the COVID-19 pandemic, such as coronavirus infection, hospitalization or death of a loved one. On the one hand, a stronger susceptibility to compensative and compulsive buying could be a reaction to these negative experiences of the pandemic; on the other hand, the increased frequency of online shopping as a result of the pandemic may be an important factor in the development of compensative and compulsive buying due to the easy implementation of purchase acts and weaker social control. To achieve the above research objectives, the German Compulsive Buying Indicator (GCBI) was used to measure susceptibility to compensative and compulsive buying. The data were obtained within three waves of the study (2010, 2019, 2022) based on a random sample of about 1,000 respondents representing statistically the general adult population. Drawing on this study, the prevalence of compensative and compulsive buying is observed at 12–19% and 2–4%. The predictors of GCBI are materialism, self-esteem, gender in all examined models and additionally age, frequency of online shopping, and experience of the COVID-19 pandemic in selected models. Although the findings related to the role of materialism, self-esteem, and gender in the prediction of GCBI reflect the results reported in the literature, the analogous conclusions about age, online shopping, and experiences with the COVID-19 pandemic are different from the established opinions. The commonly reported effect of age becomes statistically significant when the examined population is limited to Gens Y and Z. Although extensive online shopping co-exists with compensative and compulsive buying in the total population, the obtained data lead to reverse conclusions in the case of women’s subpopulation representing Gens Y and Z. The negative experience with the COVID-19 pandemic operationalised as hospitalization of a close friend predicts GCBI, but again only in the case of representatives of Gens Y and Z, especially among women. The findings show how important the creation of appropriate intervention strategies is within the consumer policy directed to representatives of the younger generations who may develop pathological buying as a response to negative experiences such as COVID-19 pandemic. The findings can inform of the goals behind therapeutic support for compulsive buyers, and implications for social work. People affected by excessive compensative or compulsive buying need to be given opportunities to build up their strengths and growth of their psychological resources towards healthy self-esteem, which seems to be the best protection against excessive compensative and compulsive buying.

## Introduction

Although Kraepelin [1] followed by Bleuler [2] described the disorder related to buying already at the beginning of the 20^th^ century, only the study by Faber, O’Guinn, and Krych [3] triggered a broader discussion which has been continued in two areas: those referring to clinical psychology and psychiatry, and those based on consumer research. The latter treat compulsive buying as an irrational consumer behaviour and they explore the socio-demographic, economic, and cultural factors of the phenomenon. Clinical psychologists and psychiatrists find compulsive buying either as a behavioural addiction or as a disorder of impulse control [4]. O’Guinn and Faber [5, p. 149] define compulsive buying as “chronic, repetitive purchasing that occurs as a response to negative events or feelings.” Although initially compulsive buyers experience purchasing acts positively, the subsequent consequences of the behaviour are negative in the psychological (feelings of guilt, weak self-esteem, distress), social (disordered interpersonal relationships), and economic (debts) sense [5]. Despite the passage of nearly 40 years since the first article on compulsive consumption was published by O’Guinn and Faber, their diagnosis of the nature of compulsive buying and its basic determinants is still valid. Contemporary researchers rely on these findings.

### Research aim

The literature review may allow to assume that the concept of compensative and compulsive buying is defined profoundly and the prevalence of the phenomenon is measured to a sufficient extent. However, this statement accurately describes the situation before the COVID-19 pandemic, because the pandemic has become a new impulse for the development of compensative and compulsive buying. What might lead to such a conclusion?

As it has been sufficiently proven by researchers, compensative and compulsive buying is a kind of reaction to negative events, situations, feelings or, more generally, to negative mental states [4–6]. Certainly, the COVID-19 pandemic should be treated as a very strong unexpected stimulus, even a shock for some people, also in the case of those who did not experience any health problems but only the social consequences resulting from lock-downs, social distancing rules, the need to wear masks, etc. [7]. The social isolation during the COVID-19 pandemic led to negative psychical states such as experienced chronic stress, anxiety feelings or mood disorders inducing compensative and compulsive buying treated by consumers as an escape from the undesired psychical states and problems of everyday life. This effect became even stronger during the pandemic social isolation because such healthier coping strategies as social interactions, physical exercise, recreational travelling etc. were limited [8].

In the case of the Polish society, this effect could have been strengthened by the social change in Poland related to the development of consumer society and secularization tendencies taking place during the recent 10–15 years. In the years 2011–2021, the purchasing power of Poles increased by 53% [9] and the infrastructure characteristics of consumer society grew from 6.5 million sq m of the shopping centres GLA to 12.3 million sq m [10]. In the same time, the consumeristic mentality based on the belief that “meaning and satisfaction in life are to be found through the purchase and use of consumer goods” [11, p. 4] diffused across the Polish society. According to the WVS [12], the ratio of adults supporting the life principle of achieving a high level of wealth grew from 25% in 2005 to 30% in 2012. The 2017 study evidenced a 72% rate of married persons characterised by at least average intensity of materialism [13]. The growth of the materialistic orientation might be supported by the secularization process. Whereas 57% of adults declared in 2005 frequent attendance at religious services (at least once a week), the ratio was 10 percentage points lower in 2017. In the same year, 18% of adults defined themselves as occasional visitors to churches or non-users of religious services. In 2005, the segment embraced 11% of Poles [12].

Difficulties faced by individuals in defining themselves may be one of the negative consequences of consumer society focused on purchase and use of goods as a source of life satisfaction. Because the strategy of identity building based on consumerism ends rather in failure, the affected individuals may consider different substitutes for their identity deficiencies. Compulsive buying might be an alternative [14].

In addition, the aforementioned lock-downs, social distancing rules, limitations of the stationary retail caused a significant growth of the e-commerce market. The share of e-commerce in the total global retail sales in 2019 amounted to the level of 13.6%, while the same indicator increased to 20.4% in 2022 [15]. The growth of the e-commerce market was a consequence of a more intensive use of online shopping which at the same time supports the development of compensative and compulsive buying to a greater extent than offline retail. It should be emphasized that the source of compensation for compulsive buyers is not the consumer good itself, but the act of purchasing. Online shopping is significantly easier to conduct without having to leave home or office. Additionally, online shopping guaranteeing greater anonymity for buyers is less socially controlled. No physical presence of sellers, no accidental unwanted witnesses of the purchase act (family members, friends, acquaintances) and easier concealment of shopping activities from household members mean that the tendency to buy compensatively or compulsively may develop faster compared to a situation when shopping can only be carried out offline. For this reason, the prevalence of compulsive and compensative buying in Poland is expected to be greater than in the years before the COVID-19 pandemic (Thesis 1).

Taking into account this new context for the development of compensative and compulsive buying, the predictive model presented in this paper includes six variables: materialism, self-esteem, gender, age, frequency of online shopping, and experience of the COVID-19 pandemic. However, the importance of the first four predictors in explaining compensative and compulsive buying has already been described in the literature, the novelty consists in the inclusion of two contextual variables ‐ frequency of online shopping and negative experiences related to the COVID-19 pandemic, such as infection of coronavirus, hospitalization or death of a loved one.

The above outlined context of the study allows the following research questions:

RQ1. Are materialism and self-esteem still important factors of compulsive buying?
RQ2. What role do demographic variables such as age, gender play in this respect?
RQ3. Is online shopping a significant factor strengthening susceptibility to compulsive buying?
RQ4. May experiences related to the coronavirus disease be considered as predictors of compulsive buying?
The above research questions will be answered based on the verification of the following hypotheses.
H1. The stronger materialism, the stronger susceptibility to compensative/compulsive buying.

An immanent feature of the materialistic orientation is the belief that satisfaction in life can be found due to the purchase and use of consumer goods [11]. This somehow forces people to participate in the consumerist race for consumer symbols of social status, although achieving success in this race is rather impossible. The satisfaction in life related to consumption is rather short-lived in the individual (the list of consumer desires is basically inexhaustible) as well as in the social sense (there are always individuals who perform better in the consumerist race) [16]. As a consequence, the people who attach more importance to the possession of consumer goods are less happy and feel more insecure [17]. Compensation in consumer goods and shopping is then an obvious way for them to improve their mood and self-perception. Finally, excessive compensation through acts of purchase might turn into compulsive buying. An important role of materialism in predicting compensative and compulsive buying has been evidenced by numerous studies [18], starting with the one by O’Guinn and Faber [5].

H2. The weaker self-esteem, the stronger susceptibility to compensative/compulsive buying.

As mentioned, excessive compensation through purchase acts may lead to compulsive buying, which is a kind of behavioural addiction. As in the case of substance addictions, low self-esteem is one of the key factors strengthening susceptibility to addictive behaviours which help to relieve tension resulting from, for example, various everyday failures. Shopping is a way to refine the poor sense of oneself and the negative frame of mind [4]. One of the sources of the tension states of oneself might be the materialistic orientation preferred by them. A weakened self-esteem might result from the specific conditions of consumer society (emphasis on having consumer symbols of social status, assessment of oneself and others through the prism of consumer goods owned, the belief that the meaning of life can be found in possession). Therefore, researchers observe a frequent coupling of materialism and self-esteem in explanations of susceptibility to compensative and compulsive buying. Similarly to materialism, self-esteem plays a significant role in predicting compensative and compulsive buying, which has been proved by numerous studies [5, 19–24].

H3. The lower age, the stronger susceptibility to compensative/compulsive buying.

Age seems to be a further important factor differentiating susceptibility to compensative and compulsive buying. Firstly, young people appreciate possession of material goods providing them with a certain amount of happiness, while a tendency to conspicuous consumption becomes weaker during the further life stages. These positive attitudes of young people towards materialistic values connected with relatively easy availability of loans (via credit cards, smartphone applications, websites etc.) could lead to the growing susceptibility of youth and young adults to compensative and compulsive buying [17]. Secondly, this relatively positive attitude of young people towards the materialistic values is supported by consumer industry which creates a symbolic value of brands. Generally, young people are more sensitive to the symbolic value of the possessed brands which may perform six basic functions: demonstration of the real or desired social status, confirmation of social competences, demonstration of identity, self-expression, creation of hedonistic experiences, and finally compensation for failures [25]. Abuse of the compensation symbolic function of consumer goods may strengthen susceptibility to compulsive buying, especially in the case of young people who use the symbolic functions of consumer goods more often. Thirdly, young people have less stable self-esteem than older people. Although building healthy self-esteem lasts a lifetime, the stages of the process can be differentiated. In most cases, self-esteem is relatively high in childhood, than it declines significantly during the adolescence phase, especially in the case of girls, and it grows gradually in adulthood. Old age is characterised again by dropping self-esteem [26]. Knowing that low self-esteem is a significant factor supporting susceptibility to compensative and compulsive buying, a declining tendency to this kind of buying should be expected along with increasing age, at least to the age interval between 60 and 70.

H4. Women show a stronger susceptibility to compensative/compulsive buying than men.

Usually, the role of gender in the explanation of susceptibility to compensative and compulsive buying is pointed in three ways. Firstly, the specific character of women’s socialization supports the development of more passive and emotional approach to manage stress and conflicts, which is based rather on legal solutions. Shopping is one way which can additionally provide social prestige in consumer society. Secondly, especially in traditional societies like in Poland, shopping is perceived as more suitable for women than for man. As a consequence, women’s compensative or compulsive buying can remain longer outside the social control, which strengthens the compulsiveness [21]. Thirdly, women care more about their appearance than men, which, under the conditions of consumer society, translates into more frequent shopping and social perception of shopping as an activity which is more natural for women than men [27, 28].

H5. The higher frequency of online shopping, the stronger susceptibility to compensative/compulsive buying.

Theoretical considerations as well as results of empirical studies indicate that online shopping could be assumed to be a further important factor differentiating susceptibility to compensative and compulsive buying. The aforementioned growth of the e-commerce market during the pandemic resulting in a considerable development of online buying instead of stationary shopping may have had an indirect impact on the spreading tendency for compulsive and compensative buying. In some way, online shopping offers more “natural” conditions to develop compensative and compulsive buying than offline shopping. Firstly, purchase acts as the source of compensation are more easily available online than offline. Thanks to computers/smartphones/other mobile devices and the Internet connection, purchase acts can be carried out anytime and anywhere. The use of the compensative function of purchase acts as a response to failures in everyday life is as easy as never before. Secondly, online shopping is more anonymous than that offline. The circumstances of offline shopping are able to render social control more effective, because the risk that strange shopping habits will be accidentally discovered by family, friends, colleagues or a seller inhibits the development of susceptibility to compensative and compulsive buying. Thirdly, online shopping might help better than shopping in stationary retail to hide compensative or compulsive buying from the closest persons. Thanks to the virtual shopping process and the use of parcel lockers to receive the ordered goods, susceptibility to compensative and compulsive buying might remain unnoticed by home dwellers longer and it can develop faster. Easy accessibility of sales points, anonymity of buyers, and circumstances that make it possible to hide strange behaviour from loved one, friends or persons from the close social environment are “classical” factors making the development of compulsive buying easier in the offline world [22]. It can be assumed that the same rules apply to the virtual world. This assumption seems to be in line with the results of previously conducted studies [29–37].

H6. People affected by the coronavirus disease or those who experienced a close person’s coronavirus disease show a stronger susceptibility to compensative/compulsive buying as compared to people without such experiences.

Yet, other potentially significant predictors of compensative and compulsive buying are negative experiences connected with the COVID-19 pandemic. Firstly, the social isolation during the pandemic brought about some negative psychical states in individuals such as chronic stress, anxiety feelings, mood disorders, weakened self-esteem, which are important factors of compensative and compulsive buying [4]. The pandemic, especially at the first stage, when the vaccines were not available at all and the mass media reported thousands of deaths a day from the coronavirus, may have strengthened people’s causal external orientation and thus the feeling that nothing depends on human will. This probably resulted in a lower level of self-efficacy, which is reported in the literature as a factor supporting susceptibility to compulsive buying [18]. As a result, behaviours such as compensative and compulsive buying might have been treated by individuals as an escape from the undesired psychical states and problems of everyday life shaped by the pandemic. Certainly, the personal experience of the coronavirus disease or an experience of the coronavirus disease by relatives or friends may have additionally fed this effect.

Secondly, the social isolation and limitations of the stationary retail has somehow forced non-users of e-commerce to discover the virtual world of shopping. This contact with the world of e-commerce may have accelerated the development of compensative and compulsive buying tendencies, especially in those who previously showed such tendencies in the offline world.

The paper is a voice in the ongoing discussion in the literature on the factors of compulsive buying, especially during/after the COVID-19 pandemic. The findings of the study expand the existing knowledge in this area and provide new insights to a better understanding of individual and social factors supporting susceptibility to compensative and compulsive buying. The present manuscript aims to contribute to the following: 1) the knowledge on the factors and severity of compulsive buying used e.g. by psychologists / psychotherapists dealing with the phenomenon, 2) the strategies of the consumer policy which prevents the spreading of socially and economically undesirable types of consumer behaviours, 3) the practise of social workers who provide support to persons suffering from compulsive buying.

### Terminological content of compulsive buying

Müller, Mitchel and de Zwaan [4] define the phenomenon as a state of desire to buy which is almost impossible to hold back. The resulting behaviour is preceded by the stage of growing tension or anxiety which can be relieved if the behaviour is conducted successfully. Initially, the shopper experiences pleasure and relief which is relatively quickly replaced by the feeling of guilt and remorse. Compulsiveness is limited to the purchasing process in this case, while the actual use of the acquired goods is not an end itself. Shopping is only a reaction to the experienced negative mood and serves to increase one’s own value, and not to satisfy the real consumer needs.

Treating compulsive buying as a type of irrational purchasing, the explanation has to take another course overcoming the difficulties springing from the post-modern paradigm which assumes that each consumer constructs their own rationality. In this sense, compulsive buying could be assessed as rational because it yields a special kind of profit for compulsive buyers, namely release of tension. The Rational Choice Theory offers the way out of the obstacle assuming that consumers conduct this behaviour from a set of alternatives which maximizes profits or minimalizes the incurred costs [38]. Therefore, compulsive buying is irrational because the costs of the behaviour are higher than the profits. Indeed, compulsive buyers may experience a relief of tension and an improved self-esteem (profits), but the potentially continuing negative consequences (costs) are much more far-reaching–distress caused by turning back remorse and feelings of guilt, loss of self-control, withdrawal symptoms, unbeneficial structure of household expenses including indebtedness, crisis of family relationships, interrupted professional career, comorbidity of behavioural or substance addiction, or even committing a crime [39, 40].

Compensation is an important dimension of compulsive buying. Assuming the latter one is an addiction to shopping, the experience of compensation through purchasing constitutes the object of the addiction. In other words, compulsive buyers always purchase compensatively; however, compensative buying may function as an autonomous purchase style preceding the stage of compulsive buying [41]. The similarity of both types of purchasing is obvious. Compensative, like compulsive buying does not primarily aim to make use of the functional utility advantages of the purchased item but to compensate for the problems occurring in different life areas [6]. In spite of the similarity, compensative buying does not equal compulsive buying in the light of the Rational Choice Theory. Although compensative buyers use purchase acts to improve their psychical state, the costs of the compensation never exceed the profits, whereas this balance is disturbed in the case of compulsive buying. Compulsive buying can be expected if the buyers consider the purchase act as a reward and lose control of their behaviour repeatedly in respect of the duration time, frequency or intensity [42].

### Prevalence

Most studies based on cross-sectional samples maximally show a 10% prevalence of compulsive buying [43]. For example, researchers from Germany observed in 2001 an 8% share of compulsive buyers in the adult population [44]. The 2010 data from Denmark proved 6% vs. 10% of compulsive and compensative buyers among 15-84-year-olds [45]. At the same time in Austria, the shares amounted to the levels of 8% and 19% [46]. A survey conducted in 2004 in the USA showed a 6% prevalence of compulsive buying among adults [47], whereas the share in Great Britain was found to be twice higher (13%) [27]. In Spain, a tendency for compulsive buying was observed in 7% of respondents belonging to the age group of 15–65 [48]. A similar share of compulsive buyers (8%) was evidenced in Eastland [49]. The results from Hungary pointed out a 2% rate of compulsive buyers among 18-64-year-olds [50], whereas the ratio among the customers of some Hungarian shopping centres achieved nearly 9% [51]. The most recent results coming from a cross-sectional study in Germany evidence a 7% share of compulsive buyers in the population aged 14 years old and more [29].

## Materials and methods

Studies on compensative and compulsive buying can be split into two basic groups: those that are carried out on typologically representative samples (then the results describe only individuals included in the sample) and those that are based on statistically representative samples (then the results can be generalized for the entire population that the sample represents) [18]. The presented study, which fits into the latter trend, was conducted in three waves in 2010 (fieldwork 14–26 June), 2019 (5–9 April), and 2022 (9–13 December) each time among about 1,000 respondents.

### Samples

Statistical representativeness of a sample requires random sampling [52]. In the case of the 2010 and 2019 studies, the simple random sampling was introduced based on the sampling frame including the Identity Personal Number owned by all country citizens. The procedure of the sampling was carried out by the State Registers Department of the Ministry of Internal Affairs. The samples were then handed over to the Market and Public Opinion Research Institute GfK, which was responsible for the fieldworks. The 2022 sampling frame based on the research online panels administered by another research company which was responsible for this stage of the fieldwork (Nationwide Research Panel Ariadna). The procedure of sampling consisted in a random selection among panellists with control of four variables (age, gender, locality class, and region of locality) to achieve a sample structure identical to the distribution of the variables at the general population level. Following the discussion in the literature, it can be assumed that the data obtained within probability-based online panels are on par with probability-based face-to-face and telephone survey [53, 54].

The 2010 and 2019 studies included each time about 1,000 participants aged 15+, while the measurement in 2022 embraced 1,093 respondents aged 18+. To improve the comparability of the particular waves, the 2010 and 2019 respondents aged 15–17 are excluded from the analysis. Because the fieldwork within the studies was carried out by external companies, the author of the paper does not have any access to information that could make it possible to identify the participants of the surveys at any stage. The socio-demographic structure of the particular samples is presented in Table 1.

**Table 1:** Sociodemographic characteristics of the sample.

	2010	2019	2022	Main Statistical Office
	*N = 933*	*N = 971*	*N = 1*,*093*	*N = 30*,*853*,*160*
**GENDER**				
**Male**	48%	48%	48%	48%
**Female**	52%	52%	52%	52%
**AGE**				
**18–24**	14%	10%	8%	8%
**25–34**	19%	18%	16%	16%
**35–44**	17%	19%	20%	20%
**45–54**	19%	15%	17%	17%
**55–64**	16%	17%	15%	15%
**65 +**	15%	21%	24%	24%
**CLASS OF LOCALITY**				
**Village**	38%	39%	38%	40%
**Town up to 20 thou. inh.**	13%	24%	13%	13%
**Town 20–50 thou. inh.**	11%		10%	11%
**Town 50–100 thou. inh.**	9%	26%	10%	8%
**Town 100–200 thou. inh.**	9%		9%	9%
**Town 200–500 thou. inh.**	9%		8%	7%
**Town above 500 thou. inh.**	11%	11%	13%	12%
**REGION**				
**South**	20%	-	21%	21%
**North-West**	15%	-	17%	16%
**South-West**	11%	-	9%	10%
**North**	16%	-	15%	15%
**Central**	10%	-	8%	10%
**East**	14%	-	16%	14%
**Mazowieckie Region**	14%	-	14%	14%

South = Małopolskie and Śląskie Regions; North-West = Lubuskie, Wielkopolskie, and Zachodniopomorskie Regions; South-West = Dolnośląskie and Opolskie Regions; North = Kujawsko-pomorskie, Pomorskie, and Warmińsko-Mazurskie Regions; Central = Łódzkie and Świętokrzyskie Regions; East = Lubelskie, Podkarpackie, and Podlaskie Regions

### Measurement

Once particular surveys had been approved by the Ethical Committee of the Institute of Sociological Sciences at the Catholic University of Lublin (decisions KEB-IS-4/2010; KEB-IS-1/2019; 15/DKE/NS/2022), fieldwork was conducted by the research companies responsible for the participants’ recruitment process including their verbal consents to take part in the surveys. The 2010 and 2019 waves were carried out using Computer Assisted Personal Interviews at the respondent’s home. The 2022 wave was conducted on the basis of Computer Assisted Web Interview. Verbal consents of the participants were ticked in the questionnaire by interviewers (CAPI) or respondents personally (CAWI). Participation in the surveys was fully anonymous.

The tendency for compensative and compulsive buying was measured by the German Compulsive Buying Indicator [55]. Respondents expressed their opinions about 16 statements using a four-point scale from 1 (“I don’t agree”) to 4 (“I totally agree”) (Q5). First, unidimensionality, reliability and normal distribution of the results on the scale were checked. The GCBI scale appeared as one-dimensional (each wave: KMO above .5; Significance of Barlett’s Test of Sphericity below .05; Eigenvalue of the first Component = 8.22 (2010), 8.52 (2019), 8.92 (2022); % of Variance = 51.37 (2010), 53.26 (2019), 55.75 (2022). In the factor analysis only one component was extracted and the solution could not be rotated. Based on the results, unidimensionality of the scale can be pointed out in the case of each wave of the survey. The scale achieved a very satisfying degree of reliability at the same time (the 2010 Cronbach’s Alpha = 0.935; the 2019 Cronbach’s Alpha = 0.941; the 2022 Cronbach’s Alpha = 0.946). It means that a high degree of internal consistency of the GCBI can be assumed for each wave of the study. The coefficients of skewness and kurtosis indicated that the distribution of the GCBI scale is approximately normal because neither of the values exceeds the interval between -1 and +1 (2010: Skewness = 0.328; Kurtosis = -0.300; 2019: Skewness = 0.295; Kurtosis = -0.284; 2022: Skewness = 0.300; Kurtosis = -0.252).

Two predictors of compulsive/compensative buying, namely self-esteem and materialism, were measured based on standardised scales. The first one, coming from Rosenberg [56], consists of 10 items which are assessed by respondents on a four-point scale from “I strongly disagree” to “I strongly agree” (Q8). After the necessary reversal of the direction of items 3, 5, 8–10, the 31-points scale shows a satisfying level of reliability (Cronbach’s Alpha = .878). Descriptive statistics of the scale are as follows: minimum value = 10; maximum value = 40; mean value = 28.89; median = 29.00; standard deviation = 5.325; Skewness = -0.036; Kurtosis = 0.473. The Richins and Dawson materialism scale [57], in Poland adapted by Górnik-Durose [58], includes 20 items which are assessed by respondents on a seven-point Likert’s scale from “I strongly disagree” to “I strongly agree” (Q7). The scale describes respondents’ susceptibility to materialism understood as a life orientation assuming that purchase, possession and consumption are the key criteria of happiness. After the necessary reversal of the direction of items 1, 3, 4, 7, 11, 15, 17, the 121-points scale shows a satisfying level of reliability (Cronbach’s Alpha = .859). Descriptive statistics of the scale are as follows: minimum value = 32; maximum value = 124; mean value = 73.80; median = 76.00; standard deviation = 14.992; Skewness = -0.260; Kurtosis = 0.297. The measurement of the remaining predictors based on the questionnaire questions about gender (Q1), age (Q2), experiences with the COVID-19 pandemic (Q10), and online shopping (Q11).

### Statistical methods

The data were analysed using SPSS.27. Univariate, bivariate, and multivariate analyses were used to describe the prevalence of compensative and compulsive buying and its relations with predictors (materialism, self-esteem, age, gender, online shopping, experience of the COVID-19 pandemic) whose selection was based on the previously formulated hypotheses. Because the respondents’ answers are described by numerical representations, it was possible to prepare descriptive statistics. The use of univariate and bivariate analyses results from the objectives of the study described in the research questions and hypotheses which can only be verified based on the quantitative data analysis methods. The stepwise multiple linear regression analysis was used to identify independent variables predicting GCBI outcomes and thus to verify the hypotheses. The advantage of this analysis is that it measures the contribution of each independent variable to the total variance, which allows determining the predictors that best explain the variance of the dependent variable within the assumed model. Additionally, the regression analysis offers the estimation of the explained variance of the whole model including all predictors [59]. As pointed out in the preceding paragraph, statistical conditions for applying the linear regression analysis have been met.

## Results

### Prevalence of compensative and compulsive buying

Following Faber and O’Guinn [41], those respondents are to be classified as compulsive buyers who achieve the result on the CBS scale of at least two standard deviations above the means. The score between one and two standard deviations allows including respondents in the segment of compensative buyers. Because the 2010 mean value on the GCBI scale equalled 31.7836 and the standard deviation 9.5383, those respondents were classified as compulsive buyers who achieved at least the result of 50 on the GCBI scale. Those respondents whose score achieved the level of 41–49 were defined as compensative buyers. This classification scheme was used in the analysis of the data from subsequent waves of the study. Table 2 presents the size of the segments.

**Table 2:** Prevalence of compensative and compulsive buying.

	2010	2019	2022
	*N = 933*	*N = 971*	*N = 1*,*093*
**Non compensative / non compulsive buyers**	83%	84%	78%
**Compensative buyers**	15%	12%	19%
**Compulsive buyers**	2%	4%	3%

The segment size of compulsive buyers remains stable between 2010 and 2022, while the share of compulsive buyers in the total population significantly increased by 7 percentage points comparing the waves before and at the end of the pandemic.

The data split into age groups confirm the above presented tendencies (Table 3). Although a growing disposition of compulsive buying cannot be concluded in any age group, the data evidence even a rapid increase of susceptibility to compensative buying.

**Table 3:** Prevalence of compensative and compulsive buying by generations.

	2019				2022			
	*N = 878*				*N = 1*,*071*			
	BB	X	Y	Z	BB	X	Y	Z
**Non compensative / non compulsive buyers**	87	82	81	82	88	77	74	59
**Compensative buyers**	10	14	15	16	12	20	20	36
**Compulsive buyers**	3	4	4	2	1	3	6	5

Gen BB = baby boomers born 1946–1964; Gen X = people born 1965–1979; Gen Y = people born 1980–1995; Gen Z = people born in 1996 and later

Gen BB does not show any significant change of their predisposition to compensative buying, whereas susceptibility of the younger generations to this buying type becomes stronger. Here, some differences are noticeable: While the 2019–2022 increase of the compensative buyers’ share in Gens X and Y is rather moderate (by 5–6 percentage points), the growth of the segment size in Gen Z exceeded the doubling of 16% and reached the value 36%.

### Predictors of compulsive buying 2022

A multiple linear regression was carried out to examine predictors of compulsive buying. On the first stage of the analysis, five variables were introduced into the model: materialism, self-esteem, age, gender, and frequency of online shopping. Because the significance level for age is unsatisfactory (p>0.05), the final model presented in Table 4 includes four remaining variables which predict almost 33% of the sample outcome variance (F(4, 1,093) = 36.411, p<0.001).

**Table 4:** Model summary for the 2022 GCBI score: Stepwise multiple regression analysis.

Step	*R*	*R²*	Adjusted *R²*	*R²* Change	*F*	*p*
**1**	0.518	0.268	0.267	0.268	399.076	<0.001
**2**	0.553	0.305	0.304	0.037	239.422	<0.001
**3**	0.560	0.313	0.311	0.008	165.442	<0.001
**4**	0.573	0.328	0.326	0.015	132.846	<0.001

Step 1: GCBI, materialism; step 2: GCBI, materialism, self-esteem; step 3: GCBI, materialism, self-esteem, gender; step 4: GCBI, materialism, self-esteem, gender, frequency of online shopping

As shown in Table 5, the final model predicts a higher GCBI score for Poles aged 18+ who are oriented materialistically (*β* = 0.444, *t* = 16.741, *p* < 0.001), show lower self-esteem (*β* = -0.216, *t* = -8.253, *p* < 0.001), and do online shopping more frequently (*β* = 0.125, *t* = 4.939, *p* < 0.001). This tendency is more characteristic of women than men (*β* = -0.090, *t* = -3.590, *p* < 0.001).

**Table 5:** Multiple regression analysis predicting 2022 GCBI score.

	Unstandardized Coefficients		Beta	*t*	*p*	95% CI		Collinearity	
	B	S.E.				Lower	Upper	Tol.	VIF
**Step 1**									
**Constant**	7.839	1.260		6.221	<0.001	5.366	10.312		
**Materialism**	0.336	0.017	0.518	19.977	<0.001	0.303	0.370	1.000	1.000
**Step 2**									
**Constant**	21.479	2.163		9.929	<0.001	17.234	25.724		
**Materialism**	0.298	0.017	0.458	17.333	<0.001	0.264	0.331	0.913	1.095
**Self-esteem**	-0.372	0.049	-0.202	-7.659	<0.001	-0.467	-0.277	0.913	1.095
**Step 3**									
**Constant**	23.551	2.231		10.558	<0.001	19.174	27.928		
**Materialism**	0.303	0.017	0.467	17.676	<0.001	0.270	0.337	0.905	1.105
**Self-esteem**	-0.369	0.048	-0.201	-7.634	<0.001	-0.463	-0.274	0.913	1.095
**Gender**	-1.740	0.493	-0.089	-3.528	<0.001	-2.708	-0.772	0.991	1.009
**Step 4**									
**Constant**	21.074	2.263		9.310	<0.001	16.632	25.515		
**Materialism**	0.289	0.017	0.444	16.741	<0.001	0.255	0.322	0.878	1.139
**Self-esteem**	-0.397	0.048	-0.216	-8.253	<0.001	-0.492	-0.303	0.900	1.111
**Gender**	-1.752	0.488	-0.090	-3.590	<0.001	-2.710	-0.795	0.991	1.009
**Frequency of online shopping**	0.937	0.190	0.125	4.939	<0.001	0.565	1.310	0.965	1.037

Table 5 presents coefficients after each predictor was added. Materialism seems to be a key variable which explains compulsive buying to the greatest extent. Self-esteem explains the GCBI score less than materialism, but still to a considerable extent comparable with gender and frequency of online shopping. Furthermore, materialism and self-esteem remain unaffected by the inclusion of gender. Basing on step 2, a growth of susceptibility to compulsive buying by 0.298 points (+/- 0.017) on the GCBI is expected with each increase of materialism by 1 point. A reverse effect is observed in the case of self-esteem. Here, an increase of self-esteem by 1 point means a loss of 0.372 points on the GCBI. Both predictors account for 30.4% of the GCBI variance.

If gender is included in the model, the effect of materialism and self-esteem on the GCBI is almost the same as in the previous models. Generally, women show a stronger susceptibility to compulsive buying than men, although the role of gender in the explanation of the GCBI in the analysed model is rather weak–if a person is a woman the result on the GCBI should increase by 1.740 points (+/- 0.493). Thereby, gender provides a very modest growth of the explained variance adding a further 0.8% to the R² coefficient (31.1%).

If frequency of online shopping is added in step 4, a growth of susceptibility to compulsive buying along with increasing materialism and declining self-esteem can be assumed on a nearly the same level as in the previous models. The tendency that women are more susceptible to compulsive buying than men is thereinafter observable, too. Meanwhile, increasing frequency of online shopping causes further growth of susceptibility to compulsive buying, although this effect is rather moderate–if frequency of online shopping increases by 1 level (on the 7-points scale from “never” to “more often than once a week”), the result on the GCBI grows nearly by 1 (+/- 0.190). Comparing the results for frequency of online shopping with materialism or self-esteem, the role of the variable in the explanation of susceptibility to compulsive buying is rather secondary. Frequency of online shopping provides a slight increase of the explained variance adding only a further 1.5% to the R² coefficient (32.6%).

Although age does not explain the GCBI score significantly in the case of the whole population, the situation changes if the subsample connecting representatives of Gens Y and Z is considered. Opposite to the whole sample, gender was excluded from the final model due to an insufficient level of significance, whereas age was included. Then, the predictors were entered in four steps starting with the variable which explains compulsive buying to the greatest extent: materialism (step 1), then self-esteem (step 2), followed by frequency of online shopping (step 3), and age (step 4), which Table 6 presents.

**Table 6:** Model summary for the 2022 GCBI score among 18-42-year-olds (generations Y and Z): Stepwise multiple regression analysis.

Step	*R*	*R²*	Adjusted *R²*	*R²* Change	*F*	*p*
**1**	0.484	0.234	0.233	0.234	130.747	<0.001
**2**	0.532	0.283	0.280	0.049	84.071	<0.001
**3**	0.544	0.296	0.292	0.014	59.714	<0.001
**4**	0.560	0.313	0.307	0.017	48.378	<0.001

Step 1: GCBI, materialism; step 2: GCBI, materialism, self-esteem; step 3: GCBI, materialism, self-esteem, frequency of online shopping; step 4: GCBI, materialism, self-esteem, frequency of online shopping, age

As shown in Table 7, the final model predicts a higher GCBI score for 18-42-year-olds representing generations Y and Z who are oriented materialistically (*β* = 0.419, *t* = 10.116, *p* < 0.001), are characterised by lower self-esteem (*β* = -0.226, *t* = -5.427, *p* < 0.001), and do online shopping more frequently (*β* = 0.137, *t* = 3.331, *p* < 0.001). This tendency is more characteristic of the younger age group than the older one (*β* = -0.133, *t* = -3.226, *p* < 0.001).

**Table 7:** Multiple regression analysis predicting 2022 GCBI score among 18-42-year-olds (generations Y and Z).

	Unstandardized Coefficients		Beta	*t*	*p*	95% CI		Collinearity	
	B	S.E.				Lower	Upper	Tol.	VIF
**Step 1**									
**Constant**	8.480	2.293		3.698	<0.001	3.972	12.987		
**Materialism**	0.332	0.029	0.484	11.434	<0.001	0.275	0.389	1.000	1.000
**Step 2**									
**Constant**	22.499	3.427		6.565	<0.001	15.763	29.235		
**Materialism**	0.301	0.029	0.440	10.521	<0.001	0.245	0.358	0.962	1.040
**Self-esteem**	-0.423	0.079	-0.225	-5.373	<0.001	-0.578	-0.269	0.962	1.040
**Step 3**									
**Constant**	19.340	3.574		5.412	<0.001	12.315	26.365		
**Materialism**	0.291	0.029	0.425	10.172	<0.001	0.235	0.348	0.947	1.056
**Self-esteem**	-0.454	0.079	-0.241	-5.757	<0.001	-0.609	-0.299	0.944	1.060
**Frequency of online shopping**	0.972	0.340	0.118	2.858	0.004	0.304	1.641	0.972	1.029
**Step 4**									
**Constant**	24.527	3.883		6.316	<0.001	16.894	32.161		
**Materialism**	0.287	0.028	0.419	10.116	<0.001	0.231	0.343	0.945	1.059
**Self-esteem**	-0.426	0.079	-0.226	-5.427	<0.001	-0.581	-0.272	0.932	1.073
**Frequency of online shopping**	1.133	0.340	0.137	3.331	<0.001	0.464	1.801	0.951	1.052
**Age**	-0.205	0.064	-0.133	-3.226	0.001	-0.330	-0.080	0.959	1.043

As previously, materialism and self-esteem appear to be key variables which explain compulsive buying to the greatest extent. Results of step 2 indicate that a growth of the GCBI by 0.301 points (+/- 0.029) is expected with each increase of materialism by 1 point. At the same time, an increase of self-esteem by 1 point means a loss of 0.423 points on the GCBI. Both predictors account for 28% of the GCBI variance. Results of step 3 allow to assume that increasing frequency of online shopping causes a further growth of susceptibility to compulsive buying, although this effect is again rather moderate–if frequency of online shopping increases by 1 level, the result on the GCBI grows by less than 1 (+/- 0.340). Frequency of online shopping provides a slight increase of the explained variance adding only a further 1.4% to the R² coefficient (29.2%). Finally, if age is included in the model, the effects of materialism, self-esteem, and the frequency of online shopping on the GCBI is similar to the previous models. Generally, the GCBI score decreases along with the growing age–an increase of age by one year results in a decline of the GCBI by 0.205 points (+/- 0.064). Again, age, like frequency of online shopping, provides a very modest growth of the explained variance adding a further 1.7% to the R² coefficient (30.7%).

Finally, does there exist any connection between susceptibility to compulsive buying and the negative experience of the COVID-19 pandemic? The latter one was operationalized as eight situations by which respondents could be hit during the pandemic: personal infection by coronavirus (35% respondents confirmed the experience), personal hospitalization due to the coronavirus infection (2%), an infected family member (41%), a family member’s hospitalization (8%), a family member’s death due to the coronavirus infection (7%), a close friend’s infection (30%), a close friend’s hospitalization (11%), a close friend’s death (10%). Unexpectedly, no type of the pandemic experience appears to be a statistically significant predictor of compulsive buying in the whole sample. The situation is quite different if the age subsample connecting Gens Y and Z is considered. It turns out that two types of the pandemic experience predict compulsive buying significantly; however, the predictive ability of the variables is rather weak. The experience of a close friend’s hospitalization due to the coronavirus infection is one of the variables. As previously, materialism was entered first (step 1), then self-esteem (step 2), followed by frequency of online shopping (step 3), age (step 4), and experience of a close friend’s hospitalization (step 5). Because pandemic experiences were measured on the nominal level, gender was excluded from the model to meet the requirements of the linear regression allowing only one nominal predictor (Table 8).

**Table 8:** Model summary for the 2022 GCBI score among 18-42-year-olds: Stepwise multiple regression analysis including the negative pandemic experience.

Model	*R*	*R²*	Adjusted *R²*	*R²* Change	*F*	*p*
**5**	0.567	0.321	0.313	0.008	40.092	<0.001

The final model presented in Table 9 predicts a strengthened susceptibility to compulsive buying for the representatives of Gens Y and Z who are oriented materialistically (*β* = 0.413, *t* = 10.006, *p* < 0.001), show lower self-esteem (*β* = -0.232, *t* = -5.580, *p* < 0.001), do online shopping more frequently (*β* = 0.132, *t* = 3.219, *p* < 0.001) and have had a negative pandemic experience due to a close friend’s hospitalization (*β* = 0.91, *t* = 2.255, *p* = 0.025). This tendency is more characteristic of the younger age group than the older one (*β* = -0.139, *t* = -3.385, *p* < 0.001).

**Table 9:** Multiple regression analysis predicting 2022 GCBI score among 18-42-year-olds having a negative pandemic experience.

	Unstandardized Coefficients		Beta	*t*	*p*	95% CI		Collinearity	
	B	S.E.				Lower	Upper	Tol.	VIF
**Step 5**									
**Constant**	25.371	3.883		6.316	<0.001	17.739	33.004		
**Materialism**	0.283	0.028	0.413	10.116	<0.001	0.227	0.338	0.941	1.063
**Self-esteem**	-0.437	0.078	-0.232	-5.427	<0.001	-0.591	-0.283	0.929	1.077
**Frequency of online shopping**	1.091	0.339	0.132	3.331	0.001	0.425	1.758	0.948	1.055
**Age**	-0.214	0.063	-0.139	-3.226	<0.001	-0.339	-0.090	0.955	1.048
**A close friend’s hospitalization due to the coronavirus infection**	3.618	1.604	0.091	-3.226	0.025	0.465	6.772	0.983	1.018

The inclusion of the variable describing the pandemic experience does not change the role of the particular predictors in the explanation of compulsive buying. As previously, materialism and self-esteem are key variables which explain the GCBI to the greatest extent. Frequency of online shopping as well as age predict compulsive buying to a lesser extent. The variable describing the pandemic experience plays rather a modest role for the explanation of compulsive buying; however, the prediction is statistically significant. A growth by 0.283 points (+/- 0.028) on the GCBI is expected with each increase of materialism by 1 point. At the same time, an increase of self-esteem by 1 point means a loss of 0.437 points on the GCBI. Adding frequency of online shopping in step 3, the GCBI grows by 1.091 point (+/- 0.339) when the predictive variable increases by 1 level. At the same time, the GCBI score decreases along with the growing age–one year more results in a decline of the GCBI by 0.214 points (+/- 0.063). Finally, if the experience of a close friend’s hospitalization due to the coronavirus infection is added, an increase of the GCBI scoring by 3.618 points (+/-1.604) can be expected. Generally, the variable provides a very modest growth of the explained variance adding a further 0.8% to the R² coefficient (31.3%).

A very interesting finding of the study is that the experience of a close friend’s hospitalization due to the coronavirus infection predicts compulsive buying only among women from Gens Y and Z, and not among men. The women’s experience of a close friend’s hospitalization is an even stronger significant predictor of compulsive buying than age. As previously, materialism was entered first (step 1), then self-esteem (step 2), followed by age (step 3), and the experience of a close friend’s hospitalization due to the coronavirus infection (step 4). As opposed to the previous scenarios, the frequency of online shopping was excluded from the model due to an insufficient level of significance (Table 10).

**Table 10:** Model summary for the 2022 GCBI score among 18–42 year-old women: Stepwise multiple regression analysis including a pandemic experience.

Step	*R*	*R²*	Adjusted *R²*	*R²* Change	*F*	*p*
**1**	0.553	0.305	0.302	0.305	61.611	<0.001
**2**	0.592	0.344	0.344	0.045	55.849	<0.001
**3**	0.605	0.356	0.356	0.015	39.635	<0.001
**4**	0.624	0.377	0.377	0.023	32.675	<0.001

Step 1: GCBI, materialism; step 2: GCBI, materialism, self-esteem; step 3: GCBI, materialism, self-esteem, age; step 4: GCBI, materialism, self-esteem, age, experience of a close friend’s hospitalization due to the coronavirus infection

As presented in Table 11, the final model predicts a strengthened predisposition to compulsive buying for women from Gens Y and Z who are oriented materialistically (*β* = 0.461, *t* = 8.026, *p* < 0.001), show lower self-esteem (*β* = -0.209, *t* = -3.668, *p* < 0.001) and had have a negative pandemic experience due to hospitalization of a close friend (*β* = 0.156, *t* = 2.802, *p* = 0.006). This tendency is more characteristic of the younger age group as compared to the older one (*β* = -0.128, *t* = -2.325, *p* = 0.021).

**Table 11:** Multiple regression analysis predicting 2022 GCBI score among 18–42 year-old women having a negative pandemic experience.

	Unstandardized Coefficients		Beta	*t*	*p*	95% CI		Collinearity	
	B	S.E.				Lower	Upper	Tol.	VIF
**Step 1**									
**Constant**	8.208	2.732		3.005	0.003	2.822	13.593		
**Materialism**	0.339	0.035	0.553	9.571	<0.001	0.269	0.409	1.000	1.000
**Step 2**									
**Constant**	20.776	4.253		4.884	<0.001	12.390	29.235		
**Materialism**	0.302	0.035	0.498	8.604	<0.001	0.235	0.375	0.962	1.040
**Self-esteem**	-0.334	0.096	-0.218	-3.776	<0.001	-0.554	-0.174	0.962	1.040
**Step 3**									
**Constant**	25.944	4.801		5.404	<0.001	16.478	35.409		
**Materialism**	0.302	0.035	0.493	8.593	<0.001	0.233	0.371	0.947	1.056
**Self-esteem**	-0.334	0.096	-0.201	-3.470	<0.001	-0.524	-0.144	0.944	1.060
**Age**	-0.185	0.083	-0.126	-2.244	0.026	-0.348	-0.022	0.972	1.029
**Step 4**									
**Constant**	27.456	4.754		5.775	<0.001	18.083	36.829		
**Materialism**	0.283	0.035	0.461	8.026	<0.001	0.213	0.352	0.900	1.111
**Self-esteem**	-0.348	0.095	-0.209	-3.668	<0.001	-0.535	-0.161	0.917	1.091
**Age**	-0.189	0.081	-0.128	-2.325	0.021	-0.349	-0.029	0.975	1.025
**A close friend’s hospitalization due to the coronavirus infection**	5.838	2.084	0.156	2.802	0.006	1.730	9.947	0.962	1.039

Unchangingly, materialism and self-esteem are the key variables which explain compulsive buying to the greatest extent. Depending on the step, a growth of the GCBI scoring by 0.283–0.339 points (+/- 0.035) is expected with each increase of materialism by 1 point. An increase of self-esteem by 1 point means a loss of 0.334–0.364 points on the GCBI (+/- 0.95–0.96). Both predictors account for 34.4% of the GCBI variance if no further variables are included into the model.

If age is added in step 3, it causes a decrease of susceptibility to compulsive buying, although this effect is rather moderate. An increase of age by 1 year goes with a decline of the GCBI scoring only by 0.185 (+/- 0.083). Contrary to that, the experience of a close friend’s hospitalization provides an increase of the GCBI scoring by 5.838 points. The inclusion of age into the model adds a further 1.5% to the explained variance in the prediction of compulsive buying (35.6%). The final model, taking into account the respondents’ experience of their close friend’s hospitalization due to the coronavirus infection, accounts for 37.7% of variance in the prediction of compulsive buying, which should be considered as a very satisfactory result.

## Discussion

As the results of three waves of the study showed, the prevalence of compulsive buying in Poland has been on a similar level since 2010. The 2–4% share of compulsive buyers in the adult population is about twice lower compared to the findings of the most cross-sectional studies carried out in the countries of West Europe or in the USA. What explains the Polish consumers’ under average susceptibility to compulsive buying? On the one hand, Poles’ purchasing power per capita is 43.4% lower compared to the EU average [9]. Although the positive correlation between income and compulsive buying has not been sufficiently proved, consumers’ better financial situation allows them to consume compensatively, which might be a preliminary stage of compulsive buying. On the other hand, different cultural conditions of the societies in West Europe or in the USA can be considered as the reasons for the relatively low ratio of compulsive buyers in Poland. Firstly, consumerism could not spread among the Poles to the degree it did in Western Europe or North America due to the historical conditions. Secondly, religious faith and practices still play an important role for at least a part of Poles independently of the relatively strong secularization processes. Because Christianity disapproves of materialism and consumerism, which are important factors of compensative and compulsive buying, religious persons may show weaker susceptibility to these kinds of consumer behaviour.

The initial thesis of the paper about the Poles’ growing susceptibility to compulsive buying is confirmed only indirectly in the light of the analyses carried out. On the one hand, numerous studies confirmed the negative correlation between age and susceptibility to compulsive buying [27, 29, 44–46, 48]. The 2010–2022 comparison of the Polish population age structure leaves no doubt as to the rapid ageing process. While the 2010 share of people aged 65+ equalled 13.6% in the total population, the current rate of the age group amounts to the level of 19.2%. According to the logic of the correlation between susceptibility to compulsive buying and age, the segment of compulsive buyers should shrink between 2010 and 2022, but it remains stable. A relative development of susceptibility to compulsive buying might be concluded.

On the other hand, the compensative buyers’ ratio increased from 12% in 2019 to 19% in 2022, especially in the case of the youngest Gen Z. It seems that consumption as a way to improve mood develops especially fast among the youngest part of the population. Treating compensative buying as a preceding stage of compulsive buying in the case of some consumers, an upcoming development of tendencies to compulsive buying in the Polish society cannot be excluded. Current representatives of Gen Z become older, but socialised susceptibility to compensative buying as a specific strategy to cope with failures of everyday life or bad mood does not disappear. It cannot ruled out that such negative consequences of the COVID-19 pandemic as social isolation and personal or indirect experience of disease accompanied by the further development of online activities strengthened young people’s susceptibility to improve the mood based on shopping and consumption.

### Materialism as a predictor of compulsive buying (H1)

In this study, an attempt is made to identify the variables that would determine GCBI scores in the examined populations. Materialism proved to be the strongest predictor of higher GCBI, which is reported in studies carried out worldwide on the samples representing general populations as well as on such specific samples as university students, adolescents, and clinical samples [18]. It was already O’Guinn and Faber [5] who pointed to the positive correlation between both variables. Otero-López et al. [60], who carried out studies among women in Spanish Galicia, indicated considerable correlations between compulsive buying and such dimensions of the full Materialism Values Scale of Richins & Dawson [57] as acquisition importance, success, and happiness. The last one turned out to be the strongest predictor of online compulsive buying in university graduated online shoppers aged 22+ living in Istanbul [61]. According to the study carried out in Eastland, only 6% of non-compulsive buyers agreed with the statement „I admire people who have expensive homes, cars and clothes”, while the ratio among compulsive buyers was 38% [49]. Dittmar [27] concluded her research results in the same way–persons valuating materialism show a stronger susceptibility to compulsive buying than persons more distanced from this life orientation. Bathia [62] found out an impact of materialism on e-compulsive buying of apparel based on the sample of 15-40-year-olds inhabiting Mumbai. Pradhan et al. [63] also proved a strengthened susceptibility to compulsive buying among respondents from India who were oriented materialistically. Some of the studies pointed out an accelerating impact of materialism on susceptibility to compulsive buying among youth and young adults. Harnish and Bridges [64] found strong correlation between Material Values Scale Short Form of Richins & Dawson [57] and Compulsive Buying Scale among US-American students. Materialism turned out to be an important predictor of compulsive buying in the adolescents coming from public school in the Midwestern USA [19]. This kind of correlation was confirmed by Sharif et al. [65], who carried out a study among Malaysian Chinese social networking sites users aged 16 to 25 years old.

The connections between materialism and compensative as well as compulsive buying under conditions of consumer society’s consumerism seem to be quite obvious. Members of the society believe that “meaning and satisfaction in life are to be found through the purchase and use of consumer goods” [11, p. 4]. It turns out that people attaching more importance to the possession of material goods are less happy and more insecure [17]. For this reason, they are more susceptible to seeking compensation in consumer goods and shopping. Finally, excessive compensative buying might turn into compulsive buying.

It is worth emphasizing at this point that the Polish society may be a particularly good ground for the development of this type of correlations between compensative/compulsive buying and materialism, which is rooted in Poland’s socialists period, especially in the 1980s. Although younger consumers do not remember this period, during the socialization and upbringing processes they internalized or they have been still internalizing very positive attitude towards brands as a means of social prestige. This cultural acceptance of brands is socially inherited from the socialist period characterised by an insufficient degree of satisfaction of the citizens’ economic needs [66] and a very high acclaim of the western pop culture as an image of freedom. Persons seeking social prestige through symbols contained in consumer goods can be characterised by a specific personal value hierarchy in which materialistic values are more or less on the top [67].

Hence, the expectation is justified that persons characterised by advanced materialism display susceptibility to compensative and compulsive buying to a greater extent than persons less attached to materialism. This expectation expressed in H1 has been clearly confirmed by the obtained data.

### Self-esteem as a predictor of compulsive buying (H2)

Self-esteem proved to be another predictor of GCBI, which is consistent with the results of other studies describing the negative correlation between self-esteem and compulsive buying. The relation was already evidenced by O’Guinn and Faber [5], who noticed a significantly lower self-esteem in compulsive buyers than in non-compulsive buyers. Studies carried out in Canada [20], Germany [21, 22], South Korea [23], Hungary [51], Italy [24] or in the USA [19] displayed the same kind of correlations. As shown above, materialism is an immanent part of consumerism which influences consumers’ beliefs that purchase and possession of material goods secure social prestige and satisfaction in life. This personal strategy leads to unavoidable failures and disappointments because a minority of consumers are able to keep up with the fashion, the appearing novelties, the newest consumer trends and the social compulsion to imitate the wealthiest. A weakened self-esteem might result from an individual’s failures in the consumer society while compensative buying and resultantly compulsive buying appear to be a simple means to improve it. This aspect can play a special role in case of the Polish society. Although the purchasing power in Poland increased by 53% between 2011 and 2021 [9], the consumer needs of Poles are satisfied by a considerable lower extent compared with consumers living in the western part of European Union. This especially applies to the younger generation which has to struggle with such problems in Poland as unavailability of mortgage loans for them, underdeveloped market of apartments for rent, or low earnings of people entering the labour market. This consumeristic disappointment reinforced by generally deteriorating consumer moods at the end of 2022 due to the high inflation and generally worse economic situation may be reflected in the low self-esteem which can cause different purchase compensation strategies [68].

This expectation of a negative correlation between self-esteem and compulsive buying expressed in H2 is confirmed by the obtained data; however, it cannot be ruled out that the predictive ability of self-esteem related to compulsive buying becomes weaker if the behaviour takes place online. The recent research points to this possibility [18]. The disappearance of the demonstrative dimension from e-compensative buying, which means that a buyer’s purchase act cannot be observed by others and the buyer’s belief about the admiration of this act by others cannot be created (it is in itself a source of compensation), might be an explanation.

### Age as a predictor of compulsive buying (H3)

Researchers also observed a stronger susceptibility to compulsive buying among younger people than among the elderly. According to Dittmar [27], compulsive buyers are younger than ordinary buyers by 8–11 years, on average. This result was confirmed in 2004 by the US-researchers who observed an interval of 9 years between the average age of compulsive buyers and other types of buyers [47]. The same kind of correlations was found by the researchers in Spanish Galicia [48], Germany [29, 44], Austria [46], and in Denmark [45]. Also, a negative correlation between age and online or offline compulsive buying was proved by researchers who conducted a tracking study in the USA during the first six months of the COVID-19 pandemic [69]. In part, this pattern is also reflected in the studies limited to representatives of young age groups. Italian researchers measured the 6.3% share of compulsive buyers in the 18-20-year-olds compared with the 14.5% rate among the 13-17-year-olds [70]. Liu et al. [71] also pointed out that age reduces compulsive buying among college students but only if age was the only predictor in the regression model. The effect of age on compulsive buying disappeared if gender, country (Germany and China) as well as the variable describing dispositional self-control were introduced into the model. An explanation for this can be sought in the simple observation that material objects and their possession as a source of personal happiness play an important role in the life of young people, while this attitude becomes weaker during the further life phases, which leads to a poorer susceptibility to compulsive or compensative buying. As Benson et al. [17] note, this materialistic orientation connected with easier availability of loans leads to the development of compensative and compulsive buying among young generations.

Unexpectedly, the results of the above presented analysis do not prove a significant prediction of the GCBI by age in the general population (H3). It does not mean that the findings are worryingly inconsistent with worldwide reported results. Age becomes a statistically significant predictor of the GCBI when the sample is limited to the representatives of Gens Y and Z (18-42-year-olds). Probably, an increasing size of the older age groups as a result of the ageing processes in the Polish society (the share of people aged 65 years and more increased from 15% to 24% between 2010 and 2022) led to the situation that the “natural” insusceptibility of older generations eliminates the statistical significance of the correlation between age and compulsive buying at the general population level.

### Gender as a predictor of compulsive buying (H4)

As expected in H5, another predictor of the GCBI is gender. Most studies pointed to a stronger susceptibility of women to compulsive buying than of men. This pattern is confirmed by studies conducted in Germany [44], Denmark [45], Austria [46], Spanish Galicia [48], Hungary [50], China [71], Pakistan [30], or recently in Germany [29] as well as by studies among adolescent consumers in Canada [20], South Korea and Poland [23], Germany and China [72]. Usually, the role of gender in the differentiation of susceptibility to compulsive buying is explained in at least three ways. Firstly, women’s socialization process is mentioned, whose specific character supports the progress of more passive and emotional ways to manage stress and conflicts based on legal solutions. One way is shopping, which is even desirable in consumer society as a source of social prestige. Secondly, the diverse socialization of the female and male roles in more traditionally oriented societies such as in Poland results in women looking after households more often than their partners. For this reason, women’s compulsive buying can be practised longer unnoticeably, which fuels the spiral of compulsiveness [21]. Thirdly, the shopping is done more often by women than by men, while positive attitudes towards shopping strengthen susceptibility to compensative or compulsive buying [27, 28].

### Frequency of online shopping as a predictor of compulsive buying (H5)

The results of the study indicate that online shopping is a further predictor of GCBI (H5); however, the role of the variable in the explanation of GCBI is more modest compared with materialism or self-esteem. The findings are in line with the results of previously conducted studies. Raab and Neuner [31] evidenced a correlation between the internet addiction and compulsive buying. The results of a recent survey on a country-wide sample in Germany prove clearly that shopping online, not necessarily being addictive, has an accelerating effect on compulsive buying [29]. Results of the studies conducted on the subsamples of the general populations lead to the same conclusions. Wang and Yang [32] pointed to a positive correlation between compulsive buying and online shopping among undergraduate students from a Taiwanese university. Bighiu, Manolica, and Roman [33] as well as Duroy, Gorse, and Lejoyeux [34] came to the same conclusions based on the studies among Romanian and French students. These findings were confirmed by the research carried out among representatives of generation Y inhabiting Bangalore in India [35] or among Pakistani students [30]. Trotzke at al. [36] confirmed clear correlations between online pathological buying and addictive Internet usage in the case of female survey participants aged 18–64 years old. The same conclusion about the speeding up effect of online shopping on women’s compulsive buying was drawn by the Chinese researchers [37].

How can this positive correlation between online shopping and susceptibility to buy compensatively or compulsively be explained? Why should online shopping enhance the development of compensative and compulsive buying to a greater extent than offline shopping? Firstly, in the case of compulsive buying, purchase acts are sources of compensation rather than a consumer good itself. It is important in the context of online shopping because purchase acts as the source of compensation are more easily available online than offline. Thanks to computers/smartphones/other mobile devices and the Internet connection, purchase acts can be carried out anytime and anywhere. The use of the compensative function of purchase acts as a response to failures of everyday life is as easy as never before. Secondly, an online shopper is more anonymous than an offline one. The circumstances of offline shopping are able to render social control more effective. The risk that compensative or compulsive buying will be accidentally discovered by persons from the close social environment inhibits the development of susceptibility to pathological consumer behaviours. Similarly to behavioural addictions, effective concealment of compensative or compulsive tendency is one of the important factors in its development [73]. Thanks to the virtual shopping process (choosing, ordering, paying) and the use of parcel lockers to receive the ordered goods, susceptibility to compensative and compulsive buying might remain unnoticed even by home dwellers longer and it can develop faster.

Thirdly, compared with offline shopping, the online marketplace offers much greater opportunities to check out what is new on the market, what new products have been introduced to the market. The search for novelties turns out to be a factor of compulsive buying [74]. On the one hand, it is rooted in the hedonistic aspect of acquiring new things [75]. On the other hand, purchase of novelties has the function of confirming the consumer’s competence on the market [25].

As a consequence, the growth of the e-commerce market during the COVID-19 pandemic caused by the increasing prevalence of online buying instead of stationary shopping may have had an indirect impact on the spreading susceptibility for compensative and compulsive buying.

### Experience of the COVID-19 pandemic as a predictor of compulsive buying (H6)

Yet other significant predictors of higher GCBI are negative experiences connected with the COVID-19 pandemic; however, the role of the predictors in the explanation of susceptibility to compulsive buying is limited. What led to these expectations? The social isolation during the pandemic brought about some negative psychical states in individuals such as chronic stress, anxiety feelings, mood disorders, depression, weakened self-esteem, which are important factors of compensative and compulsive buying [69, 76, 77]. As a result, these behaviours might be treated by individuals as an escape from the undesired psychical states and problems of everyday life shaped by the pandemic. The experience of the coronavirus disease may strengthen this effect additionally. These expectations were also supported by results of two studies. Firstly, even before the pandemic, a survey embracing female consumers who took part in the online panel of Sojump clearly showed that the perceived stress is positively associated with online compulsive buying; however, this effect becomes weaker along with growing self-esteem [78]. Secondly, a large-scaled study carried out in the United States during the first 6 months of the Covid-19 pandemic provided additional evidences of a link between the pandemic and susceptibility to compulsive buying. The application of tracking sampling every three days allowed to observe the development of susceptibility to compulsive buying over time. The conclusions are beyond doubt the following: Respondents’ susceptibility to compulsive buying grew during the first six months of the COVID-19 pandemic, especially after the CARES Act. The strongest correlation between distress caused by the pandemic and compulsive buying was reported for the respondents characterised by the highest economic position [69].

Unexpectedly, the assumptions expressed in H6 are confirmed only in part. The negative experience of the pandemic operationalised as a close friend’s hospitalization due to the coronavirus infection is a significant predictor of susceptibility to compulsive buying but limited only to representatives of Gens Y and Z (18-42-year-olds), especially among women. The commonly held opinion that the negative consequences of the social isolation during the pandemic affect more representatives of the younger age group than the older one [79–81] is confirmed in the light of the obtained data. These findings seem to be reliable in the face of the factors of compulsive buying reported in the literature. Generally, the older part of the population displays a weaker susceptibility to compulsive buying than the younger one [29, 44–46, 48, 69]. The results of the presented study indicate that the COVID-19 pandemic may have polarized the general population in this area to an even greater extent: Representatives of Baby Boomers or Gen X reject compensative buying as an antidote against discomforts of everyday life during the pandemic, while representatives of Gens Y and Z use compensative buying more willingly to improve their psychical condition. This pattern remains unchanged even when more serious consequences of the pandemic are experienced. As proved, the young people’s susceptibility to compulsive buying is strengthened by the experience of the coronavirus disease which hits close friends. This correlation becomes stronger if individuals are characterised by materialistic orientation, weak self-esteem and they do online shopping frequently. The younger age group, the stronger this phenomenon. Apparently, the threat to the health or even to the life of a close person brings a state of tension or even distress which can be relieved by compensative buying. Over time, compensative buying may become compulsive as a reaction to stress or mood swings. What is important, women aged 18–42 years old are more susceptible than men to compensative and compulsive buying on account of the experience of a close friend’s hospitalization due to the coronavirus infection, especially if three conditions are met again: materialistic orientation, weakened self-esteem and younger age. The specific way of women to deal with stress and the specific socialization to the women’s social role, especially in more traditional societies, may cause young women’s susceptibility to use of the compensation function of buying.

### Implications

The obtained data allow to draw some conclusions for psychologists/psychotherapists and social workers who help people suffering from compulsive buying, or for consumer policy coping with strategies inhibiting the prevalence of pathological buying. Similarly to other behavioural addictions, the Cognitive Behavioural Therapy is one of the most effective therapeutic activities also in the case of compulsive buying [82]. The presented findings should encourage psychologists/psychotherapists to retrospectively study the development of the patient’s compulsive behaviour, taking into account the experience with the COVID-19 pandemic in combination with the development of online consumer behaviour. The same refers to social workers. For example, the poverty of a social service client should lead to diagnosing whether their poverty is the result of compulsive shopping behaviours that developed during the pandemic. If symptoms of compulsive buying are detected, financial support for the client is insufficient but a psychotherapeutic intervention is necessary.

As evidenced by numerous studies, low self-esteem is one of the most important factors of compulsive buying. Usually, low self-esteem is rooted in childhood or in youth when authoritarian or overprotective upbringing leads to a distortion of both an individual’s autonomy and a healthy feeling of one’s own value [22]. Compensative and compulsive buying characteristic of the younger age group rather than the older one is often a response of the children and youth to the parental failures in the socialisation process, or a symptom of a youthful protest. This effect is additionally supported by the materialistic orientation characteristic of young people who usually find consumer goods and their symbolic value a relatively important way to confirm their status in the peer group and to express themselves. A low self-esteem coupled with the strong materialism pose a really high potential for the development of pathological buying. In this context, appropriate upbringing for the role of a consumer may protect young people against excessive compensative consumption in future which could turn into compulsive buying. Due to a rapid development of new technologies, deepened digitalization of everyday life, and a new role of social media in social communication parents are often not able to manage the task of consumer upbringing efficiently. Young people who are connected to the virtual world thanks to smartphones and computers [83] are constantly stimulated by marketing encouraging them to use the symbolic value of consumer goods, e.g. to compensate for the daily failures or to confirm the consumer competences [25], which can turn into compulsive buying. Moreover, recent studies show a correlation between smartphone addiction and online compulsive buying in the case of Gen Z [84]. Special educational programmes showing children and youth as well as their parents the harmful effects of pathological buying (and other behavioural addictions including addiction to smartphones, computers, social media or more generally to new technologies) could be a solution supporting the insufficient upbringing process in the area of buying and consumption. These special educational programmes should be implemented in the form of new, special lessons in primary schools (in Poland first eight years of education) when children already have their own money and develop consumer attitudes. In secondary schools, the issues related to the nature, conditions and effects of compensative and compulsive buying could be included in the current lessons of entrepreneurship. In addition, activities related to informing about the dangers caused by pathological buying should be undertaken by consumer rights ombudsmen, whose task in Poland is to provide consumers with advice and legal information. Currently, consumer rights ombudspersons mainly provide assistance to consumers at the initiative of the latter. The scope of consumer rights ombudspersons’ activities should be expanded to include preventive measures in the form of educational strategies addressed to children as well as adolescent and adult consumers, e.g. popular science articles in print and online media, information via social media, information brochures distributed by local government units (in Poland, there is an ombudsperson in each poviat), lectures in senior clubs. These solutions would be in line with the most important tasks of consumer policy divided into three main areas–consumer education, consumer information, and consumer protection [85].

### Study limitations

Some limits of the study should be openly mentioned. Firstly, the obtained data could have been influenced by the effect of social desirability bias, first of all in the case of the older generation. This effect is observable in the social research, especially devoted to the topic of addiction if the method of questionnaire is introduced. Then, the conclusions about compulsive buying are based on the declarations, not on the actual behaviour. The appearance of the social desirability bias effect is greater in the case of older than younger people because representatives of the older age groups find different life values important and tend to yield to social control. Secondly, the respondents were questioned about the pandemic experiences from the past while the measurement of susceptibility to compulsive buying is related to the general pattern of behaviour. It is not known whether compensative and compulsive buying was a direct reaction to the negative experiences such as a close person’s coronavirus disease. Hence, conclusions can be made only about the co-occurrence of the memories and current susceptibility to compensative or compulsive buying. In addition, the study is not longitudinal; hence, the conclusions are all the more possible about the coexistence of the analysed variables but not about any causal relations.

## Conclusions

The initial thesis 1 about Poles’ stronger current susceptibility to compensative and compulsive buying compared with the period before the COVID-19 pandemic is confirmed only in part. Although the share of compulsive buyers did not grow between 2010 and 2022 actually, the size of the compensative buyers’ segment increased significantly, especially among the representatives of Gen Z. This growth from 16% to 36% of compensative buyers’ share in Gen Z might be an indicator of spreading compulsive buying in future. Additionally, inferences about the development of compulsive buying in the Polish society might be drawn indirectly. Assuming that susceptibility to compulsive buying becomes weaker along with age, actually the aging process of the society should cause the effect of compulsive buyers’ shrinking share. The stable 2010–2022 share of compulsive buyers in the total population (2–4%) does not justify such conclusions.

The aim of the presented study was to answer four research questions and to verify six hypotheses. The first research question (RQ1) concerned the role of materialism and self-esteem in explaining susceptibility to compensative and compulsive buying. As expected, both variables play a crucial role in this area. On the one hand, the stronger materialistic orientation, the stronger susceptibility to compulsive buying. On the other hand, the best protection against compulsive buying is a healthy self-esteem. Thus, the hypotheses assuming an increase in susceptibility to buy compensatively or compulsively with an increase in materialistic orientation (H1) and weakening self-esteem (H2) were confirmed.

The second research question (RQ2) refers to the role of sociodemographic variables such as age and gender in predicting susceptibility to compensative and compulsive buying. It was hypothesized that susceptibility to buy compensatively or compulsively would decrease with age (H3) and that the share of compensative and compulsive buyers would be higher among women than among men (H4). Surprisingly, age does not explain susceptibility to compulsive buying considering the general population, which might be an effect of the rapid ageing process in the Polish society. However, when the sample is limited to 18-42-year-olds representing Gens Y and Z, age begins to play a role in the prediction of compulsive buying. The correlation is in line with the worldwide reported results: The younger age groups, the stronger susceptibility to compulsive buying of their representatives. However, this correlation is not strong. Also gender is not a strong predictor of susceptibility to both types of consumer behaviours. Generally, women show a stronger susceptibility to compensative and compulsive buying than men, although the role of gender in the explanation of compensative and compulsive buying is rather modest. The verification of hypotheses 3 and 4 shows that younger people and women tend more often to buy compensatively and compulsively than older people and men, but neither factor is crucial for the development of the susceptibility.

The next research question (RQ3) concerns the role that online shopping plays in explaining susceptibility to compensative and compulsive buying. It is assumed that the increasing frequency of online shopping goes hand in hand with a stronger susceptibility to compensative and compulsive buying (H5). Indeed, the increasing frequency of online shopping goes along with growing susceptibility to compensative and compulsive buying, although this effect is rather moderate. Comparing the results for frequency of online shopping with materialism or self-esteem, the importance of the variable for the explanation of susceptibility to compensative and compulsive buying is rather secondary, although slightly greater than in the case of age and gender.

The fourth research question (RQ4) refers to the COVID-19 pandemic as a potential factor strengthening compensative and compulsive buying. It was hypothesized that people affected by the coronavirus disease or those who experienced a close person’s coronavirus disease show a stronger susceptibility to compensative and compulsive buying as compared to people without such experiences (H6). Unexpectedly, a negative experience of the COVID-19 pandemic appears as a statistically significant predictor of compulsive buying in the general population. If the analysed population is limited to representatives of Gens Y and Z, the situation is completely different. The negative experience of the COVID-19 pandemic operationalised as a close friend’s hospitalization due to the coronavirus infection predicts susceptibility to compensative and compulsive buying to a similar extent as frequency of online shopping. This effect is even stronger if the analysed population is limited to the female representatives of Gens Y and Z. Then the variable describing negative experiences with the pandemic, such as a close friend’s hospitalization due to the coronavirus infection, predicts susceptibility to compensative and compulsive buying more strongly than age and only slightly weaker than self-esteem.

To summarise, the strongest susceptibility to compulsive buying can be expected among materialistically oriented young online shoppers characterised by low self-esteem who experienced a close friend’s hospitalization due to the coronavirus infection during the pandemic. This effect is even stronger when the sample of 18-42-year-olds is limited only to women. The only difference is that online shopping does not play any role in the explanation of women’s susceptibility to compulsive buying. Thereby, the previously assumed hypotheses 1–6 describing the correlations between compulsive buying and materialism, self-esteem, age, gender, online shopping, and negative pandemic experiences are confirmed at least in part. The findings can provide information on the goals behind the therapeutic support for pathological buyers, and implications for social work with them. The findings also prove that the pathological buying might be a growing serious problem among the youngest consumers and we urgently need more studies on this issue.

## Supporting information

S1 Questionnaire Questionnaire PL. (DOCX)

S2 Questionnaire Questionnaire ENG. (DOCX)
