# Supplementary material for: Pathological buying on the rise? Compensative and compulsive buying in Poland in the pre- and (Post-)pandemic times
Source: PLoS One. 2024 Mar 21;19(3):e0298856. doi: 10.1371/journal.pone.0298856 (PMC10956761; doi:10.1371/journal.pone.0298856)
Supplement: S1 Questionnaire — (DOCX) [file pone.0298856.s001.docx]

**KWESTIONARIUSZ**

**P1. Płeć**

**P2. Wiek**

**P5.** **Poniżej znajduje się kilka stwierdzeń dotyczących kupowania i sytuacji z tym związanych. Przy każdym stwierdzeniu proszę zaznaczyć, w jakim stopniu ono pasuje lub nie pasuje do Pana(i)?**

SKRYPTER: ROTACJA STWIERDZEŃ.

*W wierszach:*

1) Czasami bez szczególnej przyczyny nagle czuję, że muszę wyjść z domu i pójść na zakupy

2) Czasami mam wyrzuty sumienia, gdy coś sobie kupię

3) Czasami widzę coś i czuję, że muszę to kupić

4) Czasami zauważam, że coś mnie popycha do tego, by iść na zakupy

5) Często kupuję coś tylko dlatego, że jest tanie

6) Często kupuję coś, gdyż po prostu mam ochotę na kupowanie

7) Często mam poczucie, że jakąś rzecz muszę bezwzględnie mieć

8) Często nie mam odwagi pokazać kupionych rzeczy innym osobom, gdyż mogliby mnie uznać za osobę nierozsądną

9) Często po zakupie jakiejś rzeczy pytam samego siebie, czy rzeczywiście ten zakup był taki ważny

10) Gdy idę przez miasto albo przez centrum handlowe/ sklep, czuję silną potrzebę, by coś sobie kupić

11) Gdy mam pieniądze, czuję, że muszę je wydać

12) Jestem raczej rozrzutny(a)

13) Katalogi sprzedaży wysyłkowej/ strony sklepów internetowych są dla mnie dużą pokusą, po ich obejrzeniu najczęściej wysyłam zamówienie

14) Robię zakupy, by uciec od niemiłej codzienności i się zrelaksować

15) Zdarzało mi się często kupić coś, czego potem w ogóle nie używałe(a)m

16) Zdarzało mi się często kupić coś, na co właściwie w ogóle nie mogłe(a)m sobie pozwolić

*W kolumnach:*

1. Całkowicie nie pasuje do mnie
2. Raczej nie pasuje do mnie
3. Raczej pasuje do mnie
4. Całkowicie pasuje do mnie

**P7. Proszę pomyśleć przez chwilę o rzeczach, które do Pana(i) należą i zastanowić się, jaką pełnią one funkcję w Pana(i) życiu, jakie mają dla Pana(i) znaczenie. Następnie proszę zaznaczyć na skalach, w jakim stopniu zgadza się Pan(i) z każdym z podanych poniżej stwierdzeń. Nie ma tu odpowiedzi dobrych lub złych, chodzi nam o to, by Pan(i) najlepiej określić swoje prawdziwe przekonania i opinie.**

SKRYPTER: ROTACJA STWIERDZEŃ.

*W wierszach:*

1) Posiadanie dóbr materialnych ma dla mnie mniejsze znaczenie niż dla większości ludzi, których znam
2) Uważam, że jedno z najważniejszych osiągnięć życiowych polega na zdobyciu wysokiej pozycji materialnej
3) W sensie materialnym mam wszystko, czego potrzebuję, by cieszyć się życiem
4) Zasób posiadanych dóbr nie jest oznaką sukcesu życiowego
5) Sądzę, że był(a)bym szczęśliwszy(a), gdybym miał(a) lepsze rzeczy, niż aktualnie posiadam
6) Lubię mieć przedmioty, które robią duże wrażenie na innych
7) Nie zwracam szczególnej uwagi na to, co inni ludzie posiadają
8) Czasem bardzo doskwiera mi to, że nie mogę mieć wszystkich tych rzeczy, które chciałbym posiadać
9) Przedmioty, które mam, pozwalają mi dobrze czuć się wśród moich przyjaciół i znajomych
10) Posiadanie pewnych przedmiotów materialnych może dodawać pewności siebie
11) Zgromadzone dobra materialne nie świadczą o moim sukcesie i powodzeniu życiowym lub ich braku
12) Posiadanie dóbr materialnych może zapewnić poczucie wolności i niezależności
13) Usilnie dążę do zdobycia dóbr i przedmiotów materialnych, o których marzę
14) Mam wiele takich rzeczy, których nie dałoby się zastąpić innymi
15) Fakt posiadania lub nieposiadania pewnych dóbr nie wpływa na moją samoocenę
16) Cieszy mnie już samo posiadanie pewnych rzeczy
17) Nie byłbym bardziej zadowolony, gdybym mógł pozwolić sobie na kupowanie droższych rzeczy
18) Gromadzenie dóbr i pieniędzy jest dla mnie przyjemnością
19) Ludzie, którzy posiadają kosztowne przedmioty, domy, samochody itp., budzą we mnie podziw i respekt.
20) Luksus to jedna z najważniejszych wartości w moim życiu

*W kolumnach:*

1. Zdecydowanie nie zgadzam się
2. Nie zgadzam się
3. Raczej nie zgadzam się
4. Trudno powiedzieć

5. Raczej się zgadzam

6. Zgadzam się

7. Zdecydowanie zgadzam się

**P8.** **Poniżej znajdują się różne stwierdzenia, które odnoszą się do Pana(i) przekonań o sobie. Proszę wskazać, w jakim stopniu zgadza się lub nie zgadza się Pan(i) z każdym z tych stwierdzeń, wybierając jedną z czterech możliwych odpowiedzi. Proszę postarać się określić to, co naprawdę Pan(i) sądzi. Liczą się tylko szczere odpowiedzi.**

SKRYPTER: ROTACJA STWIERDZEŃ.

*W wierszach:*

1) Uważam, że jestem osobą wartościową przynajmniej w takim samym stopniu, co inni

2) Uważam, że posiadam wiele pozytywnych cech

3) Ogólnie biorąc jestem skłonny(a) sądzić, że nie wiedzie mi się

4) Potrafię robić różne rzeczy tak dobrze, jak większość innych ludzi

5) Uważam, że nie mam wielu powodów, aby być z siebie dumnym (dumną)

6) Lubię siebie

7) Ogólnie rzecz biorąc, jestem z siebie zadowolony (a)

8) Chciał(a)bym mieć więcej szacunku dla samego siebie

9) Czasami czuję się bezużyteczny(a)

10) Niekiedy uważam, że jestem do niczego

*W kolumnach:*

1. Zdecydowanie nie zgadzam się
2. Raczej nie zgadzam się
3. Raczej zgadzam się
4. Zdecydowanie zgadzam się

**P10.** **Jakie są Pana(i) doświadczenia z chorobą COVID-19?**

1) Byłem/ byłam zakażony(a) koronawirusem

2) Byłem/ byłam hospitalizowany(a) z powodu zakażenia koronawirusem

3) Członek rodziny był zakażony koronawirusem

4) Członek rodziny był hospitalizowany z powodu zakażenia koronawirusem

5) Członek rodziny zmarł z powodu zakażenia koronawirusem

6) Bliski znajomy był zakażony koronawirusem

7) Bliski znajomy był hospitalizowany z powodu zakażenia koronawirusem

8) Bliski znajomy zmarł z powodu zakażenia koronawirusem

9) Trudno powiedzieć

**P11.** **Jak często kupuje Pan(i) coś przez internet – w jakimś sklepie internetowym, czy za pośrednictwem platformy takiej jak Allegro?**

1) Przeciętnie, częściej niż jeden raz w tygodniu

2) Około jeden raz w tygodniu

3) Jeden–dwa razy w miesiącu

4) Kilka razy w roku

5) Przeciętnie, jeden raz w roku

6) Jeszcze rzadziej

7) Nigdy
