# Supplementary material for: Pathological buying on the rise? Compensative and compulsive buying in Poland in the pre- and (Post-)pandemic times
Source: PLoS One. 2024 Mar 21;19(3):e0298856. doi: 10.1371/journal.pone.0298856 (PMC10956761; doi:10.1371/journal.pone.0298856)
Supplement: S2 Questionnaire — (DOCX) [file pone.0298856.s002.docx]

**QUESTIONNAIRE**

**Q1. Gender**

**Q2. Age**

**Q5.** **There are a few statements related to buying and situations connected with it. Please tick at each statement to what extent does each of statements fit you or not?**

*SCRIPTER: Rotation of the statements*.

*In rows:*

1. I often have an unexplainable urge, a sudden and spontaneous desire, to go and buy sometimes in a store

2. At times, I have felt somewhat guilty after buying a product

3. There are times when I have a strong urge to buy

4. I sometimes feel that something inside of me pushed me to go shopping

5. Often I buy something just because it is cheap

6. Often I buy something because simply I feel like buying

7. Often I have a feeling that I absolutely must have an item

8. There are some things I buy that I do not show to anybody for fear of being perceived as irrational in my buying behaviour

9. Often I ask myself after a purchase of an item if the purchase was really so important

10. As soon as I walk down streets or I enter a shopping centre, I have an irresistible urge to go into a shop to buy something

11. When I have money, I cannot help but spend part of the whole of it

12. I am rather free-spending

13. I am one of those people who often responds to direct mail offers/ websites of online stores

14. For me, shopping is a way of facing the stress of my daily life and of relaxing

15. I have often bought something which I do not use at all

16. I have often bought a product that I did not need, while knowing I had very little money left

*In columns:*

1. I disagree

2. I rather disagree

3. I rather agree

4. I totally agree

**Q7. Please think for a moment about the things that belong to you and consider what they do in your life, what they mean for you. Then, please mark on the scales to what extent you agree with each of the following statements. There are no right or wrong answers here, we want to identify best your true beliefs and opinions.**

*SCRIPTER: Rotation of the statements*.

*In rows:*

1. I put less emphasis on material things than most people I know
2. Some of the most important achievements in life include acquiring material possessions
3. I have all the things I really need to enjoy life
4. The things I own say a lot about how well I’m doing in life
5. I’d be happier if I could afford to buy more things
6. I like to own things that impress people
7. I don’t pay much attention to the material objects other people own
8. It sometimes bothers me quite a bit that I can’t afford to buy all the things I’d like
9. The things I own allow me to feel well among my friends
10. The possession of some material goods might breathe confidence into myself
11. The material goods do not evidence the life success
12. The possession of material goods might provide sense of freedom and independency
13. I strive persistently to acquire material goods and objects that I dream about
14. I have many such things that could not be replaced by others
15. Having or not having certain goods does not affect my self-esteem
16. I enjoy just having certain things
17. I wouldn’t be any happier if I owned nicer things
18. Collecting goods and money is a pleasure for me
19. I admire people who own expensive homes, cars, and clothes etc.
20. I like a lot of luxury in my life

*In columns:*

1. I definitely disagree
2. I disagree
3. I rather disagree
4. Difficult to say

5. I rather agree

6. I agree

7. I definitely agree

**Q8.** **Below is a list of statements dealing with your general feelings about yourself. Please indicate how strongly you agree or disagree with each statement.**

*SCRIPTER: Rotation of the statements*.

*In rows:*

1. I feel that I’m a person of worth, at least on an equal plane with others

2. I feel that I have a number of good qualities

3. All in all, I am inclined to feel that I am a failure

4. I am able to do things as well as most other people

5. I feel I do not have much to be proud of

6. I take a positive attitude toward myself

7. On the whole, I am satisfied with myself

8. I wish I could have more respect for myself

9. I certainly feel useless at times

10. At times I think I am no good at all

*In columns:*

1. Strongly disagree
2. Rather disagree
3. Rather agree
4. Strongly agree

**Q10.** **What are your experiences with COVID-19?**

1) I was infected with coronavirus

2) I was hospitalised due to coronavirus infection

3) A family member was infected with coronavirus

4) A family memebr was hospitalised due to coronavirus infection

5) A family member has died of the coronavirus infection

6) A friend was infected with coronavirus

7) A friend was hospitalised due to coronavirus infection

8) A friend has died of the coronavirus infection

9) Difficult to say

**Q11.** **How often do you buy something via Internet in an online store or based on such platforms as Allegro?**

1) More often than once a week on average

2) About once a week

3) Once-twice a month

4) A few times a year

5) Once a year on average

6) More seldom

7) Never
